# Pleistocene climate variability in eastern Africa influenced hominin evolution

**DOI:** 10.1038/s41561-022-01032-y

**Published:** 2022-09-26

**Authors:** Verena Foerster, Asfawossen Asrat, Christopher Bronk Ramsey, Erik T. Brown, Melissa S. Chapot, Alan Deino, Walter Duesing, Matthew Grove, Annette Hahn, Annett Junginger, Stefanie Kaboth-Bahr, Christine S. Lane, Stephan Opitz, Anders Noren, Helen M. Roberts, Mona Stockhecke, Ralph Tiedemann, Céline M. Vidal, Ralf Vogelsang, Andrew S. Cohen, Henry F. Lamb, Frank Schaebitz, Martin H. Trauth

**Affiliations:** 1 Institute of Geography Education, University of Cologne, Cologne, Germany; 2 Department of Mining and Geological Engineering, Botswana International University of Science and Technology, Palapye, Botswana; 3 School of Earth Sciences, Addis Ababa University, Addis Ababa, Ethiopia; 4 Research Laboratory for Archaeology and the History of Art, University of Oxford, Oxford, UK; 5 Large Lakes Observatory and Department of Earth & Environmental Sciences, University of Minnesota Duluth, Duluth, MN USA; 6 Department of Geography and Earth Sciences, Aberystwyth University, Aberystwyth, UK; 7 Berkeley Geochronology Center, Berkeley, CA USA; 8 Institute of Geosciences, University of Potsdam, Potsdam, Germany; 9 Department of Archaeology, Classics and Egyptology, University of Liverpool, Liverpool, UK; 10 MARUM Center for Marine Environmental Sciences, University of Bremen, Bremen, Germany; 11 Department of Geoscience, Eberhard Karls Universität Tübingen, Tübingen, Germany; 12 Senckenberg Centre for Human Evolution and Palaeoenvironment, University of Tübingen, Tübingen, Germany; 13 Department of Geography, University of Cambridge, Cambridge, UK; 14 Institute for Geography, University of Cologne, Cologne, Germany; 15 LacCore/CSDCO, Department of Earth and Environmental Sciences, University of Minnesota, Minneapolis, MN USA; 16 Unit of Evolutionary Biology/Systematic Zoology, University of Potsdam, Potsdam, Germany; 17 Institute of Prehistoric Archaeology, University of Cologne, Cologne, Germany; 18 Department of Geosciences, University of Arizona, Tucson, AZ USA; 19 Department of Botany, School of Natural Sciences, Trinity College, University of Dublin, Dublin, Ireland

**Keywords:** Evolutionary ecology, Palaeoclimate, Limnology

## Abstract

Despite more than half a century of hominin fossil discoveries in eastern Africa, the regional environmental context of hominin evolution and dispersal is not well established due to the lack of continuous palaeoenvironmental records from one of the proven habitats of early human populations, particularly for the Pleistocene epoch. Here we present a 620,000-year environmental record from Chew Bahir, southern Ethiopia, which is proximal to key fossil sites. Our record documents the potential influence of different episodes of climatic variability on hominin biological and cultural transformation. The appearance of high anatomical diversity in hominin groups coincides with long-lasting and relatively stable humid conditions from ~620,000 to 275,000 years bp (episodes 1–6), interrupted by several abrupt and extreme hydroclimate perturbations. A pattern of pronounced climatic cyclicity transformed habitats during episodes 7–9 (~275,000–60,000 years bp), a crucial phase encompassing the gradual transition from Acheulean to Middle Stone Age technologies, the emergence of *Homo sapiens* in eastern Africa and key human social and cultural innovations. Those accumulative innovations plus the alignment of humid pulses between northeastern Africa and the eastern Mediterranean during high-frequency climate oscillations of episodes 10–12 (~60,000–10,000 years bp) could have facilitated the global dispersal of *H. sapiens*.

## Abstract (web summary)

Over the past 620,000 years, three distinct phases of climate variability in eastern Africa coincided with shifts in hominin evolution and dispersal, according to an analysis of environmental proxy records from a core collected in the Chew Bahir basin of Ethiopia.

## Main

**Fig. 1:**
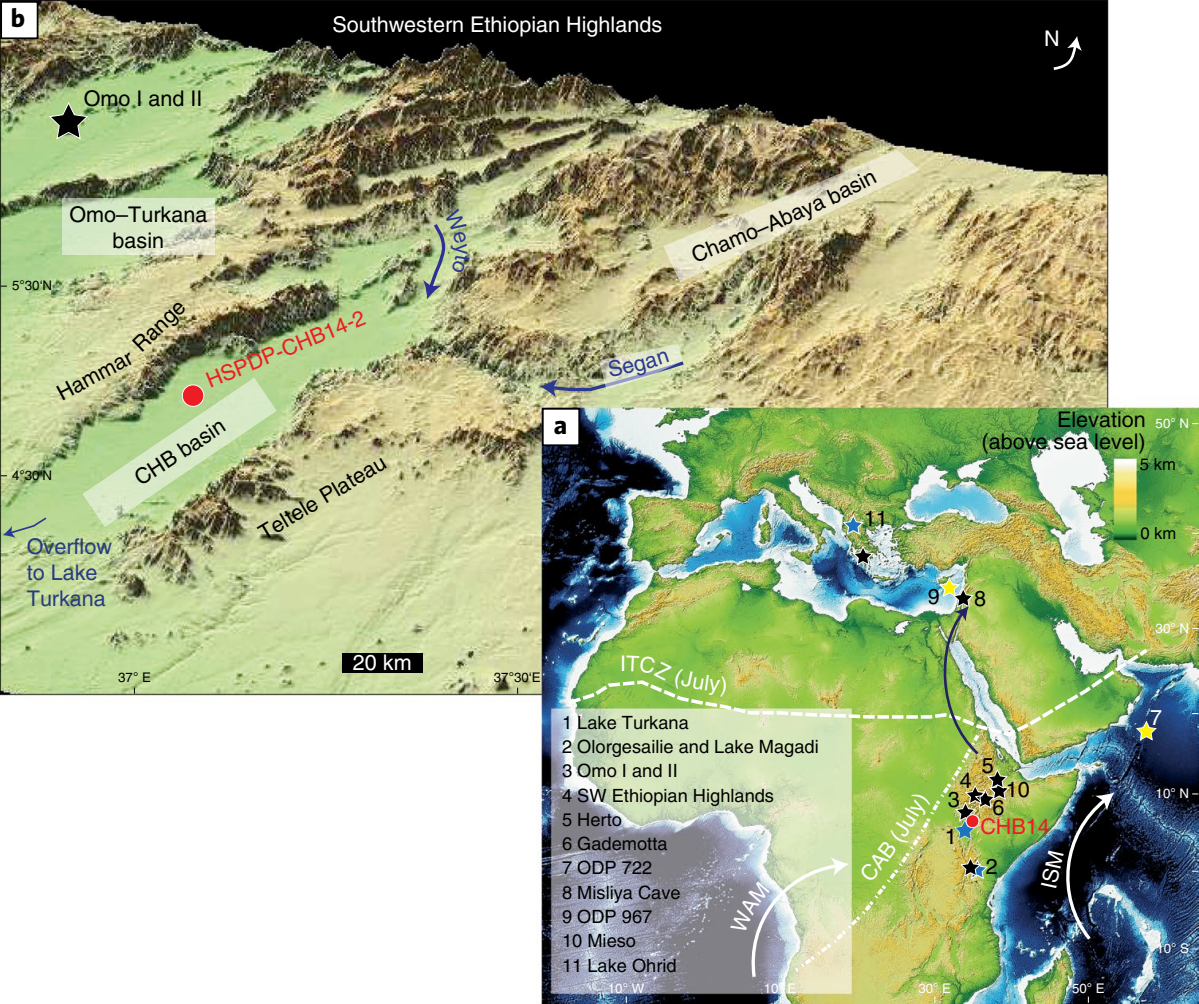
Map of CHB basin and its location. **a**, Location of the CHB basin within the East African Rift System with major climatic influences, key archaeological and palaeoanthropological sites, marine and terrestrial records and the main northern route for later dispersals of *H. sapiens* as discussed in the text. **b**, Topographic map of the CHB basin showing the drainage network, core site of HSPDP-CHB14-2 and sites in the vicinity. ISM, Indian summer monsoon; WAM, West African monsoon; CAB, Congo Air Boundary; ITCZ, intertropical convergence zone.

Eastern Africa during the Middle–Late Pleistocene offered a wide range of habitats, and deposits of this age are rich in human fossils and archaeological remains^1–4^. Hypotheses seeking to explain links between climate and human origins are difficult to test because both climate records and traces of early human populations are often incomplete or poorly dated^3–5^. Encouraged by discussion of possible climate-evolution linkages, the Hominin Sites and Paleolakes Drilling Project (HSPDP) was established in 2008, with deep drilling campaigns in 2013–2014^6^. One component of HSPDP, the Chew Bahir Drilling Project (CBDP), collected two ~280-m-long cores from Chew Bahir (CHB), a playa lake in southern Ethiopia (4° 45' 40.5″ N, 36° 46' 1.0″ E) (Fig. 1)^7,8^, covering the past ~620,000 years (620 kyr)^9^, which includes the time frame of the emergence of *H. sapiens* in Africa^6,10,11^. While there is a general consensus that the physical, cognitive and cultural evolution of *H. sapiens* in Africa developed multiregionally^11,12^, the CHB record provides an environmental window with which to view the eastern African part of this history^6^. The CHB coring site is situated near key archaeological and palaeoanthropological sites, such as the Omo-Kibish (~90 km west of CHB) (Fig. 1)^2,6,13^. In this Article, we provide one of the first continuous environmental contexts from a proven habitat of early *H. sapiens* enabling evaluation of hypotheses linking climate change with increasing versatility and innovation in the hominin lineage and the history of human dispersal within and out of Africa^11,12,14^ (see Methods for details of coring and analysis of the CHB sediments).

## CHB core stratigraphy and environmental history

**Fig. 2:**
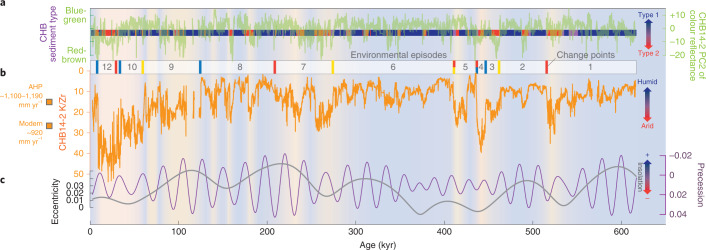
Environmental change at CHB. **a**, Relation between distribution of CHB sedimentary facies 1 and 2 and variation of CHB sediment colour (second principal component (PC2)). High PC2 scores of the colour reflectance values correspond to blue-green sediment colours (of facies 1 sediment), whereas low PC2 scores correspond to red-brownish hues (facies 2)^15^. **b**, CHB14-2 K/Zr record with high values indicating arid climate conditions (note inverted scale) and interpreted environmental episodes 1–13, with intervals showing similar trends in the mean (blue bars), standard deviation (yellow bars) and trend (red bars) of the K/Zr time series according to the results of the change-point analysis (Extended Data Fig. 3 and Extended Data Tables 1 and 2). **c**, Orbital forcing^51^. Background shading refers to simplified hydroclimatic conditions (red, arid; blue, humid). Humidity marker on the left *y* axis indicates modelled African Humid Period (AHP) moisture level (20–30% precipitation increase) for CHB catchment (~1,100–1,190 mm yr^–1^ catchment mean) in relation to modern-day precipitation (~920 mm yr^–1^ catchment mean)^8^.

**Extended Data Fig. 1:**
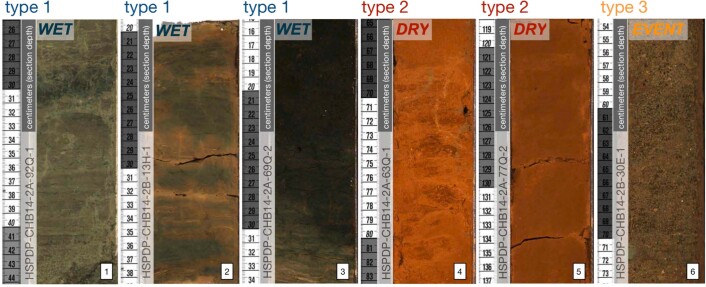
Selected facies from CHB14-2 cores that are representative of different environmental conditions. [1–3] are clays, calcareous silty clays and clay-rich silts, some show horizontal laminations; these facies represent characteristic colors and properties of type 1 facies [WET]; [4,5] are typical of type 2 facies [DRY], comprising calcareous silt and clayey silt; [6] with unordered sand deposits, is an example for type 3 facies [EVENT].

**Extended Data Fig. 2:**
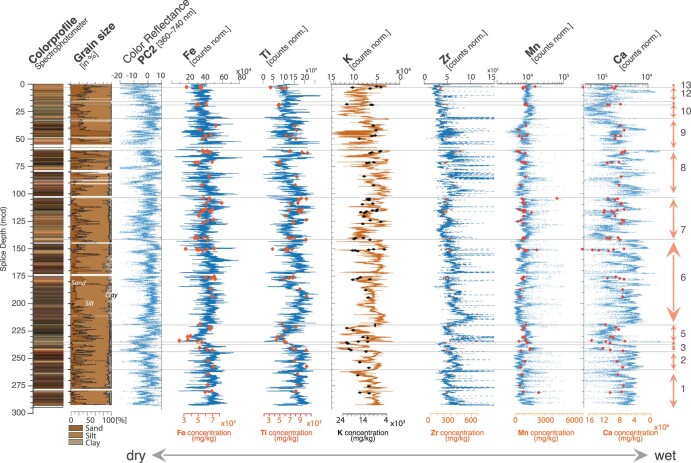
Summary stratigraphy of composite core HSPDP-CHB14-2 and results of chemical and physical sediment analyses. Columns from left to right: Core color profile (MSCL- spectrophotometer), grain sizes in %, PC2 of the color reflectance (ref. ^15^) and scanning XRF results (5 mm resolution) in normalized counts report to the upper x-axis (blue plots and red curve) and are compared to results of quantitative XRF analyses (concentrations in mg/kg) that report to the lower x-axis (red and black squares). Elemental variations from left to right: iron (Fe), titanium (Ti), potassium (K) (note inverted scale), zirconium (Zr), manganese (Mn), calcium (Ca) (note inverted scale). Episodes on the right axis correspond to environmental episodes in Fig. 2. Proxy values oriented to the right indicate wet conditions; proxy values oriented to the left are indicative for dry conditions.

Regional climate variability of the past ~620 kyr has been recorded in shifts between two principal lacustrine sedimentary facies of the CHB cores (Fig. 2 and Extended Data Figs. 1 and 2). Facies 1 is a blue-green or grey clayey silt, reflecting anoxic conditions at the sediment/water interface and/or higher levels of organic matter. This is associated with poorly ventilated bottom water of the lake during episodes of high water level and a wetter climate^15^. Facies 2 is light brown and reddish-brown silt. The brown colour results from the formation of an abundance of oxidized iron at the oxygenated sediment surface associated with episodes of lower lake levels or exposed lake floor during drier climate conditions^15^. Intercalation and mixing of sediment types occur at transitions reflecting changes in the hydrology of the lake and its catchment (see Methods for details of sediment colour data).

These moisture regime changes at CHB are captured by varying potassium (K) bulk-sediment concentration, determined by X-ray fluorescence (XRF) core scanning^7^. In saline systems, K concentrations in sediment can reflect the degree of authigenic alteration of smectite to illite, responding sensitively to the hydrochemistry (alkalinity and salinity) of palaeolake and pore waters, which in a closed lake basin corresponds to changes in water depth^8,16^. High K in CHB, fostered by an increase in alkalinity and salinity under lowering lake levels during dry climate episodes, is further enhanced by octahedral Al-to-Mg substitutions in clay minerals and a resulting layer charge increase^16^. Part of the variation in K concentration, however, is also attributable to weathering and transport of detrital material into the basin, linked to humidity (physical and chemical weathering, mobilization and transport) in addition to aridity (reverse weathering). To correct the K values for the effects of dilution by non-clay materials and transport, we normalized K by the zirconium (Zr) values (K/Zr), a proxy for detrital input reflecting the input of clastic material^13^. Thus, assuming that K/Zr is a faithful recorder of hydroclimatic changes at CHB, we applied a hydro-balance model to estimate the precipitation associated with the changes in K/Zr. During the last African Humid Period (~15–5 kyr) with low K/Zr ratios (<10), an increase of 20–30% annual precipitation (+200 mm yr^–1^) would have been required for the palaeolake to rise to the outflow level (±45 m above present-day levels)^8^. Modern conditions of ~900 mm yr^–1^ and a seasonal shallow lake show K/Zr ratio of ~28 corresponding to the sediment surface (Fig. 2). This allows us to estimate an order-of-magnitude range for the hydroclimatic changes of the past ~620 kyr.

**Extended Data Fig. 3:**
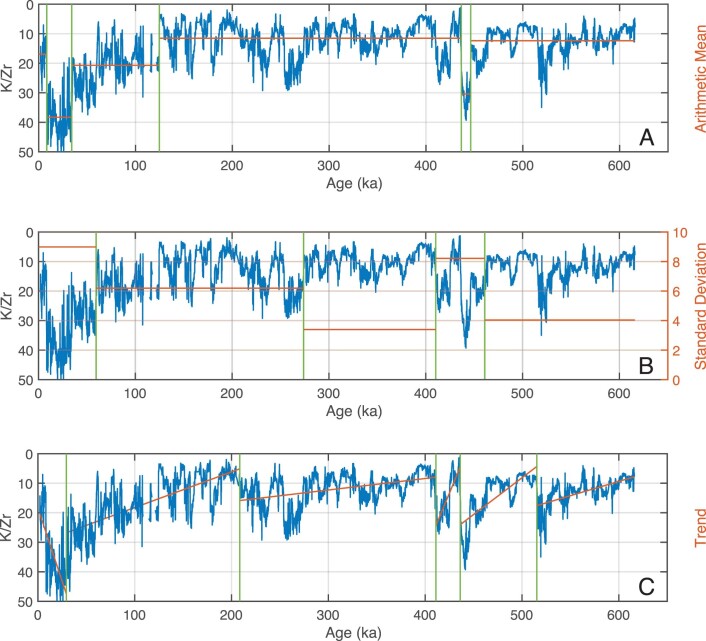
Change point analysis of the CHB14-2 K/Zr record. Change points in the statistical measures in the K/Zr record from CHB cores were determined to structure the long CHB-14-2 K/Zr record using the MATLAB-based function *findchangepts* contained in the MATLAB Signal Processing Toolbox. We run *findchangepts* for the arithmetic mean (Supplementary Fig. 3a), standard deviation (Supplementary Fig. 3b) and trend (Supplementary Fig. 3c) for a maximum of five important changes. All change points were applied to the CHB14-2 K/Zr time series, dividing the record into 13 episodes.

On the basis of a change-point analysis of the K/Zr time series, we can identify 13 environmental episodes in the CHB sequence (Fig. 2, Extended Data Fig. 3 and Extended Data Tables 1 and 2; see Methods for details of the application of the change-point analysis). The oldest half of the record (~620–275 kyr), comprising episodes 1–6, is characterized by relatively stable wet conditions during episodes 1, 2 and 6, interrupted by abrupt shifts between hydrological extremes in episode 3 and especially episodes 4 and 5. The upper–middle part of the record (~275–60 kyr; episodes 7–9) is characterized by an important increase in variability, in both frequency and magnitude. While episodes 7 (~275–210 kyr) and 8 (~210–125 kyr) remain relatively wet, the sequence, beginning with episode 8, exhibits a long-term drying trend with intensifying relatively low-frequency oscillations, culminating in significantly drier conditions from episode 9 (~125–60 kyr) onwards. This part of the sequence exhibits periodic wet–dry cycles on an ~20 kyr timescale^13^. The youngest part of the record (after ~60 kyr; episodes 10–13) is characterized by an unprecedented increase in variability with high-frequency alternations^13^. This period includes the most arid and rapidly oscillating climate of the entire record during episodes 11 (~35–30 kyr) and 12 (~30–10 kyr), but with a strong reduction in sediment accumulation rate, potentially a hiatus, between ~30 and 12 kyr (ref. ^9^). A reversal towards wetter conditions occurs with the abrupt onset of episode 13 at ~10 kyr, followed ultimately by a shift to modern-day, persistently dry climate conditions (Fig. 2 and Extended Data Fig. 3).

## Controls of eastern African climate change

**Extended Data Fig. 4:**
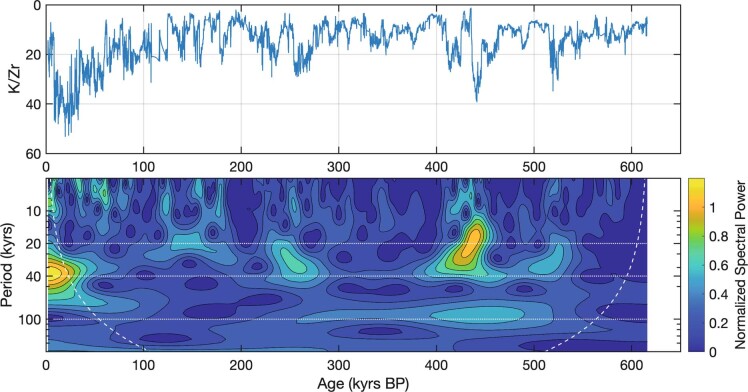
Wavelet power spectrum for the K/Zr record from CHB14-2 cores. The data were interpolated to an evenly spaced time axis and analyzed using the *cwt* function contained in the MATLAB Signal Processing Toolbox. The white line marks the cone of influence, which is used to mark the area where edge effects occur in the wavelet transformation. The wavelet power spectrum clearly shows significant periodicities at 100 ka (especially between 400–500 ka BP) and between 15–40 ka (especially between 400–500 ka BP and between 150–300 ka BP).

The long-term trends in the K/Zr record show an anticorrelation with eccentricity (~125 and 400 kyr cyclicity) and a correlation with precession (~21 kyr and ~11 kyr half-precessional cyclicity)^15,17,18^, while the influence of obliquity (~40 kyr cyclicity) is weak or absent^19^ (Fig. 2 and Extended Data Fig. 4). This is in agreement with the observation of strong orbital controls on long-term climate change in the tropics^17^, in which orbital precession, modulated by eccentricity, paces insolation variation and in turn the extension and intensity of the summer monsoon rains over northern Africa and the Arabian Peninsula^18,20^. Mediterranean influences have also been suggested as a driver of increased winter rains during phases of enhanced seasonality in insolation^13^. During periods of muted orbitally controlled variation of insolation (430–360 kyr and 120–0 kyr), other factors, such as greenhouse gas and ice-sheet forcing, which have been suggested as controls on precipitation over eastern Africa^21^, could also have had increased influence at CHB (see Supplementary Note 3 for additional details on hydroclimatic controls).

As a second possible driver of CHB’s hydroclimate through time, wet–dry variations in the record appear to be in phase with sea surface temperature fluctuations recorded in Ocean Drilling Program (ODP) Site 722^19^ that reflect the intensity of coastal upwelling in the Arabian Sea and therefore the strength of the summer monsoon. Thus, amplified wet phases in southern Ethiopia may have been enhanced by moisture advection and a stronger summer monsoon in phase with orbital forcing^22^ (Supplementary Fig. 3). By contrast, variations in greenhouse gas concentration, such as atmospheric CO_2_ (ref. ^21^), show a much weaker (at least in some time intervals) similarity to the K/Zr moisture index from CHB with possible exceptions during the eccentricity minima between ~430–360 kyr and ~120–0 kyr (Supplementary Fig. 3). Such an exception is particularly well expressed at ~430 kyr and the transition into Marine Isotope Stage (MIS) 11 and could potentially relate to the simultaneous Mid-Brunhes Event (MBE). The MBE signals a profound shift in the global carbon cycle, which led to warmer interglacials with higher atmospheric CO_2_ levels starting with MIS 11. The rapidly rising CO_2_ levels at the MIS 12/11 transition could have facilitated an increase in precipitation at CHB. A link between the MBE and the eastern African moisture regime has also been recognized at Lake Magadi, Kenya^23^ (see Supplementary Note 3 for details on forcing and glacial boundary conditions).

The regional impact of climate fluctuations inferred from the CHB record can be assessed by comparison with other continental records in Africa and with marine records from the Indian Ocean and the Mediterranean Sea. Moisture fluctuations that are in phase point to potential inter-regional climate connections and possible common underlying causes. The wet–dry index from ODP Site 967^24^ from the eastern Mediterranean Sea, dominated by the Nile River outflow, shows a strong similarity to wet–dry shifts recorded at CHB^25^, arising from the close geographical proximity of the Blue Nile and CHB catchments (Fig. 1 and Supplementary Fig. 3). Thus, when wet climate prevailed in both catchments, the moisture signal was transmitted by the Nile into the Mediterranean Sea, explaining the correspondence of the hydrological signal between CHB and the eastern Mediterranean. However, during eccentricity minima, the coupling between CHB and ODP Site 967 appears to lessen, supporting our hypothesis that eccentricity is the major driver of moisture availability at CHB. Crucially, when comparing the northeastern Mediterranean site of Lake Ohrid^26^ with CHB (Supplementary Fig. 3), we note some coincident pulses of elevated humidity during eccentricity minima. This may have resulted from a southward shift of winter jets and storm tracks in the Mediterranean region during glacial episodes and periods of reduced Northern Hemisphere insolation^17,26^.

## Implications for humans and their habitats

**Fig. 3:**
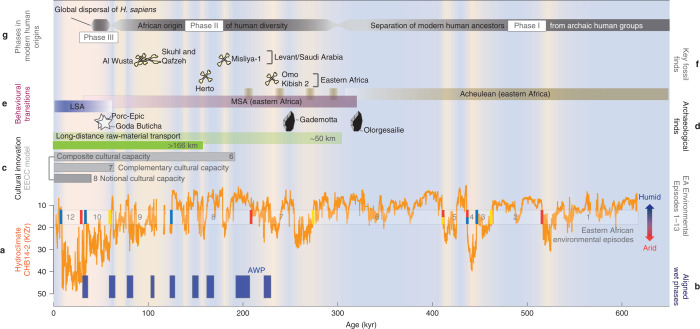
Comparison of CHB record and identified eastern African environmental episodes with key milestones and transitions in modern human origins. **a**, CHB climate record (aridity proxy K/Zr) and 13 episodes of inferred habitat transformation (Fig. 2); **b**, Wet phases in CHB that align with wet pulses in northwestern Africa^24^, and the Mediterranean^26^ (Supplementary Fig. 3) as potential time windows for favourable migration routes out of Africa; AWP, aligned wet phases **c**, Innovation, comprising social, technological, symbolic and cultural evolution; evolution and expansion of cultural capacities (EECC) model^41^ with stage 6 comprising technological augmentations, stage 7 with complementary cultural capacity, for example, bow and arrow, and stage 8 with notional cultural capacity, for example, notional concepts and beliefs (see Supplementary Note 4 for detailed information); long-distance raw-material transport, indicative of formation of social networks^42^. **d**, Key archaeological finds discussed in the text: earliest MSA occurrence in Ethiopia at Gademotta^29^ and in Kenya at Olorgesailie^3^ and early symbolic artefacts at Porc-Epic^49^, Goda Buticha^50^. **e**, Behavioural transitions: Acheulean–MSA transition^31^; MSA–Later Stone Age (LSA) transition^46^. **f**, Key *H. sapiens* fossil finds in eastern Africa and the Levant/Saudi Arabia as discussed in the text; **g**, Simplified key phases of modern human origin adapted from ref. ^11^.

Hydroclimatic variability of the past ~620 kyr recorded in the CHB cores resulted in multiple profound environmental transformations in an important region for hominin evolution. This variability included several long, stable humid episodes (~10^4^ to >10^5^ yr) that may have created favourable conditions for hominins and a series of shorter episodes (~10^3^ to 10^4^ yr) (Extended Data Table 2) during which intense environmental stress was caused by a combination of aridity and rapidly fluctuating conditions, partially superimposed on long-term aridification trends (Fig. 2). While both the climatic and archaeological records have inevitable chronological uncertainties, a comparison on evolutionary timescales offers a useful opportunity to examine possible linkages (Fig. 3).

Favourable habitats for hominins in eastern Africa can be deduced for episodes 1, 2 and 6, marked by relatively long-lasting stable and humid conditions at CHB (Extended Data Table 2). These relatively stable, wet conditions between ~620 and 275 kyr were interrupted by arid pulses in episodes 3–5. The aridification observed at the onset of episode 3, and especially the abrupt and extreme climatic oscillations observed during episodes 4 and 5, are likely to have caused vicariance due to fragmentation of habitats, shifts in population ranges and local extinctions of hominin populations. Adaptations to novel, dramatically transformed local environments among small, reproductively and culturally isolated populations probably stimulated the appearance of the many geographically and anatomically distinct hominin groups^5,11^ found within this period (~620–275 kyr). While some of this variation may have been reduced by subsequent dispersal during more favourable periods, much persists beyond 275 kyr, where it forms the foundation for the morphological diversity seen in early *H. sapiens* populations^5,12,27^. Within Ethiopia, the distinct morphs of the Omo I and Omo II fossils provide a snapshot of this diversity^28^.

During episodes 7–9 (~275–60 kyr), we infer that a pattern of cyclicity transformed habitats in eastern Africa. This interval shows significantly increased variability, both in frequency and in magnitude of climate swings. This pronounced variability is superimposed on an aridification trend from ~210 kyr that accelerates after ~120 kyr. The onset of this period of high variability coincides with dates for the earliest Middle Stone Age (MSA) occurrence in Ethiopia at Gademotta (279 ± 2 kyr (ref. ^29^)), reflecting a technological trajectory similar to that seen at Olorgesailie^3^ (Kenya), where a total replacement of Acheulean by MSA technologies had been completed at least by ~320 kyr (refs. ^3,30^). While Gademotta shows no Acheulean elements^29^, Ethiopian localities such as Herto and Mieso show a late persistence of the Acheulean^31,32^. Taken together, the Kenyan and Ethiopian records suggest that prolonged periods of variability such as those evident in the CHB record may have driven the gradual replacement of Acheulean by MSA technologies and the spread of *H. sapiens*. The earliest *H. sapiens* fossils in eastern Africa (Omo I: >233 ± 22 kyr (ref. ^33^); Herto BOU-VP-16/1: 156 ± 7 kyr) (refs. ^1,31,34^) also appear during this period; their geographically distinct mosaics of ancestral and derived features are consistent with population isolation caused by habitat fragmentation^35^.

The cyclic climatic variability of episodes 7–9 is likely to have paced the expansion and contraction of habitats utilized by MSA populations^13^. While contractions probably caused hominins to retreat to refugial zones (such as the southwestern Ethiopian Highlands)^13,36^, potentially resulting in allopatric differentiation, expansions could have enabled renewed contact and the spread of beneficial variants^10^. Such mechanisms have the potential to maintain vital genetic heterogeneity^35^, promote cultural innovation and diversity^37,38^ and enhance rates of cumulative cultural evolution^39^. Environmental shifts at precessional timescales are slow enough to be mitigated by geographic range shifts; the short-term climate flickers demonstrated at CHB, however, were too rapid (<10^3^ yr) to be entirely accommodated by relocation, particularly in cases where areas of viable habitat were discontinuous. Local extinctions were therefore probably among populations unable to adapt in situ. Populations of slowly reproducing species such as *H. sapiens* are unlikely to be able to respond to such rapid changes by genetic adaptation alone. Instead, they may have responded plasticly, by utilizing their cognitive abilities to support social learning, with adaptation being primarily cultural^40^. Although climatic changes and their populational effects are unlikely to be the sole drivers of cultural adaptation, several critical cognitive and societal developments mark this interval as a key phase of technological and social innovation^41,42^ that would have equipped *H. sapiens* with unprecedented adaptability. Evidence of more varied tool kits, long-distance transport and symbolism^3,29,42^ appear during periods of rapid environmental perturbation, demonstrating more developed cultural capabilities among MSA hominins^3^. Such innovations emerge in situ but subsequently equip populations to disperse into novel environments^43,44^. Both technological and social innovations could have buffered early *H. sapiens* from the impacts of severe environmental changes^10,42^.

Some populations of *H. sapiens* were able to disperse beyond Africa before 60 kyr (refs. ^11,45^), although it is not currently possible to identify their distinct geographical origins. Several pronounced wet phases at CHB during episodes 7–9 align with humid pulses in northeastern Africa^13,25^ and the Mediterranean^26^, perhaps opening favourable migration routes out of Africa on a roughly north–south axis along the East African Rift System and into the Levant^11,45^. One possibility is that early dispersals involved populations dispersing in line with expansions or shifts of suitable habitat^15^. As cultural innovations accumulated during episodes 7–9, however, they provided *H. sapiens* populations with the means to expand beyond their native habitat^14,37,44^, with this expansion ultimately leading to the global dispersal of modern humans^11^.

Episode 10 (~60 kyr) begins with a brief but pronounced return to humid conditions, followed by a crucial shift to unprecedented high-frequency oscillations and an increase in aridity. This critical episode encompasses the beginning of the major *H. sapiens* dispersal but also witnesses the further accumulation of innovations within eastern Africa. The transition from the MSA to the Later Stone Age is protracted^46^ but is signalled by increases in smaller tools such as backed microliths and by bipolar (anvil-assisted) flaking^47,48^. Symbolic innovations such as shell beads (at Porc-Epic^49^) and engraved ostrich eggshell (at Goda Buticha^50^) occur within Ethiopia during episode 10. Thus, although many of the innovations facilitating widespread dispersal were in place before episodes 10–12, the extreme environmental fluctuations observed during this interval continued to act as a motor for indigenous cultural change.

In summary, the results from the CHB core record suggest that shifting of environmental conditions during the past ~620 kyr could have played a major role in human biological and cultural evolution. This shaped adaptations and provided environmental opportunities facilitating modern human dispersal out of Africa.

## Methods

### CHB coring site and sediment cores

In November–December 2014, the CBDP retrieved duplicate sediment cores from the western margin of the CHB basin in the southern Ethiopian Rift, to 278.58 metres below surface (core HSPDP-CHB-2A) and 266.38 metres below surface (core HSPDP-CHB-2B)^6,52^. CHB is a deep tectonic, endorheic basin that episodically held a palaeolake hydrologically open during pronounced wet phases, with an outflow to the south at +45 m relative to the current playa surface^7,8^. The two coring sites are ~20 m apart, being situated in proximity (~3 km distance) to the western shore of the playa with shallow but extensive alluvial fans (up to 20 km by 10 km), draining the Hammar Range (Fig. 1b)^53^. The playa surface at the coring site represents the modern surface of an at least 3-km-deep sediment fill of the deep tectonic basin, as seismic surveys for petroleum exploration by Tullow Oil Company showed^6,7,52^.

### Core processing and inter-core correlation

The core was described, measured, logged, sampled and spliced according to protocols at the US National Lacustrine Core Facility^6,52^, where archive halves are also permanently curated. Low-resolution (5 cm) multi-sensor core logging (MSCL[-S])^54^, including magnetic susceptibility, gamma-ray density and p-wave velocity, non-contact electrical resistivity and natural gamma radiation, provided first data for initial core correlation and enabled the parallel processing and description of core sections upon opening. High-resolution line-scan images^54^ were taken with a Geotek MSCL-CIS digital line-scan core imager for both cores HSPDP-CHB-2A and HSPDP-CHB-2B directly after length-wise splitting and cleaning of the core sections. Initial Core Description using the PSICAT software with a HSPDP description key provided a visual lithological description, including smear slides analyses at boundaries of changing lithologies (composite stratigraphical column comprising all lithological descriptions is available at 10.17605/OSF.IO/M8QU5). On all split core halves, MSCL (MSCL-XYZ)^54^ in 5 mm resolution was applied, comprising ~55,000 data points for magnetic susceptibility and colour reflectance spectrophotometry (greyscale and 360–740 nm bands in 10 nm steps)^15^ (Extended Data Fig. 2) to determine the physical sediment properties for the entire length of the CHB record. The two CHB cores 2A and 2B were spliced together on a common-depth scale based on visual characteristics (images and open core), sedimentological data and physical properties (MSCL data and Initial Core Description) and metadata, using the IODP standard content and format (splice and affine tables). For subsequent small-scale refinements of the herein used version 3.0 of the splice^54^, chemical sediment characteristics have also been used. The resulting composite core HSPDP-CHB14-2 comprises 292.87 m (Extended Data Fig. 2) with a core recovery of ~90% (splice and affine tables along with the core metadata, images and MSCL data are available at 10.17605/OSF.IO/M8QU5).

### Sampling

Subsamples for all analytical parameters were retrieved according to sampling plans along the composite core, following a routine 32 cm sampling increment for the whole core, with higher resolution for high-interest intervals and opportunistically for proxies/samples that require specific material compositions (for example, optically stimulated luminescence dating and biomarkers), resulting in ~14,000 discrete sediment samples.

### Sediment types

Besides the two contrasting sedimentary facies 1 and 2 (Fig. 2 and Extended Data Fig. 1) and transitional types that can be associated with intermediate compositions between the two, we can differentiate a further minor facies. Sedimentary facies 3 is coarse grained, poorly sorted and medium brown, and the associated layers are distinctly separated from underlying and overlying deposits. We infer that these sediments were deposited during discrete depositional events such as short but intense floods. This generated high sediment rates within a short time (hours–days), but without sustainably affecting the hydrological system entirely. During the deposition of these sediments, Lake Chew Bahir was generally shallow or seasonally dried out, and evaporation was exceeding precipitation. When vegetation cover on the adjacent slopes of rift shoulders and alluvial fans was reduced, material could be eroded more easily^7,55^.

### Grain size

Grain sizes were measured for the composite core using a 32 cm interval (743 discrete samples) at the laboratory of the Geographic Institute, University of Cologne. The determination of grain size was performed in 116 channels from 0.04 to 2,000 μm using a laser diffraction particle size analyser (LS 13320 Beckmann CoulterTM). The calculation of grain-size raw data was performed using the Fraunhofer optical model. Before analysis, organic and carbonate content was removed using 15% H_2_O_2_ and 10% hydrochloric acid (HCl), respectively. Before measurements, the samples were treated with sodium pyrophosphate (Na_4_P_2_O_7_) to avoid aggregation. Mineralogical results suggested that the removal of in situ precipitates such as gypsum that can potentially influence grain-size results was not required due to rare occurrences in the mineral suite of CHB samples. Grain-size parameters are based on Folk and Ward^56^ and were calculated by GRADISTAT software version 8^57^. Granulometric information can be used to provide insights into erosional and transport mechanisms in the CHB catchment, sedimentation dynamics of detrital material and provenance of detritus (Extended Data Fig. 2)^13,55,58,59^. Grain-size distributions in the CHB record have been used to infer sediment transport processes. Analyses of the data showed that coarser grain sizes in particular (up to coarse sand) are transported primarily by the extensive alluvial fans aggrading from the western rift shoulder into the CHB basin^7,13,60^. Those alluvial fans become increasingly active during generally drier climate conditions in a landscape with reduced vegetation^7,60^. Elevated proportions of the finer fractions through the record indicate a relatively high water level of the palaeolake and are typically deposited during humid conditions (such as episodes 1, 2, 6 or 8). In several intervals, clay-enriched beds alternate with sand layers. We interpret intervals with these frequently interchanging layers as reactivation products of the alluvial fans. These layers suggest extreme rainfall with reduced vegetation cover in the catchment during intermittent dry periods. Silt-sized particles are much more abundant throughout the record and dominate beside sand the uppermost 43 metres composite depth (~0–130 kyr)^13^. High amounts of sand and silt with the absence of clay and fine silt can be indicative of long-lasting dry conditions.

### Sediment colour and colour reflectance data

Variations in sediment colour were determined by colour reflectance spectrophotometry in 5 mm resolution as part of the MSCL analyses^15,58^. Sediment colour may be the result of the primary mineralogical constituents and their diagenetic changes reflecting oxic versus anoxic conditions at sediment deposition^13,61,62^. The ratio of haematite versus Ti-magnetite, for example, would be a typical example of minerals represented in the CHB catchment^16,53,55^ responding to oxidation of the detrital Ti-magnetite. Dissolved iron (Fe) and iron-hydroxides may originate from the volcanic bedrocks in the higher eastern boundary of the CHB basin, the Teltele Plateau and from the northeastern part of the catchment, where Cenozoic volcanic rocks are exposed to weathering (Fig. 1 and Extended Data Fig. 2)^7,16^. The reduced minerals and the resulting colour reflectance retain their diagenetic lake phase signature during the absence of oxygen, while more sediments deposited on top finally seal the previously deposited sediments from chemical changes of the lake waters. In both cases, darker sediment colours are an indication of changes of the bottom-water ventilation in the course of water-level fluctuations, with blue-green colours during high lake levels (associated with humid conditions) and reddish-brown colours during low lake levels (associated with arid episodes)^7,16^ (Fig. 2 and Extended Data Fig. 2). Principal component analyses of the colour reflectance values^15^ helped to unmix the environmental factors controlling sediment colour and to increase the signal-to-noise ratio, as well as to facilitate interpretation of the multivariate dataset. The first principal component (PC1) shows similar loadings for all colour bands and is interpreted as the total reflectance (overall brightness of the image) and hence not used for further analyses. Instead we use the PC2 (3.4% of the total variance) with positive loads within the short wavelengths (blue reflected light) as an indicator of wetter conditions in the basin (Fig. 2 and Extended Data Fig. 2)^15^.

### X-ray fluorescence (XRF) analysis

#### X-ray flourescence (XRF) core scanning

In this study, 84 representative samples along the CHB composite core were selected for the determination of quantitative elemental concentrations (mg kg^–1^) throughout the core. Sample selection covers areas of high and low peaks as well as in higher resolution (32 cm) transitions between high and low XRF counts (counts per second). The comparison between quantitative XRF data measured on discrete samples and the Itrax core-scanning data facilitates the assessment and potential correction of possible matrix effects. To assess possible variations in the elemental concentration through time, the effect of varying grain sizes, surface effects and water saturation have to be determined by comparing quantitative with the semi-quantitative results. All discrete samples were dried and ground to powder fraction, pressed with equal pressure (2 × 25 kg) into sample holders. All quantitative measurements were performed at the Institute of Geochemistry at MARUM Bremen using a PANalytical Epsilon 3XL energy-dispersive XRF equipped with a rhodium tube, several filters and an SSD5 detector. A calibration based on certified standard materials (GBW07309, GBW07316, MAG-1) was used to quantify elemental counts. Three reference samples were used, indicating that all shown results are well within the limit of detection. The quantitative XRF concentrations in mg kg^–1^ correlate strongly with the semi-quantitative line scans in counts per second, with Pearson correlation coefficients varying among 0.91 (Fe), 0.80 (Ti), 0.80 (K), 0.94 (Zr) and 0.91 (Ca) for elements within the limits of detection. Matrix effect correction^63^ and numerical calibration^64,65^ for strongly offset scanning results is not applicable given the good agreement between quantitative and semi-quantitative datasets (Extended Data Fig. 2).

XRF core scanning, at 5 mm resolution, was performed at the Large Lake Observatory of the University of Minnesota to determine the elemental sediment composition. An Itrax core scanner with a chromium (Cr) tube as radiation source, a tube voltage of 30 kV, current of 30 mA and scanning time of 10 s was used. Data were normalized to compensate for aging of the Cr tube^66^ by weekly measurements of a set of US National Institute of Standards and Technology Standard Reference Materials (SRMs 1646a, 2711a, 2586, 1944 and 2702) and coherence scattering. In 2018, three additional standards were developed for better calibration of high-carbonate sediments by dilution of SRM 2711a with reagent grade CaCO_3_. Pressed powder samples of the SRMs were measured at a resolution of 0.2 mm along their axes, resulting in 25 readings for each of the chemical elements as well as for coherence scattering.

The CHB cores were measured between 16 April 2016 and 12 October 2018, including 21 SRM runs. A weekly correction factor was determined for each element by dividing long-term average values of SRM measurements by the week’s measurements. The values for coherence scattering have been corrected accordingly. The element counts were divided by the normalized coherence scattering and multiplied by the correction factor for the specific week. A small number (24) of sections were re-run in July–August 2019 because they displayed system offsets in light elements that were not fully corrected by the initial normalization process. Quality flags between 0 and 2 have been continuously attributed sub-centimetre wise to the composite core material and have then been applied to each measured data point (= 0.5 cm resolution) for both MSCL and XRF datasets. The quality flags also consider section breaks, with possibly disturbed section ends, cracks, large concretions, coring artefacts or larger voids.

For the CHB basin, the best environmental proxy indicating aridity is the hydrochemically controlled potassium content (K) and the K/Zr ratio (Fig. 2)^7,16,18,55^. Authigenic processes such as illitization and zeolitic alteration with increasing alkalinity and salinity have been described for CHB to increasingly alter weathering silicates such as smectites into authigenic phases, at the expense of smectite abundances^16,60,67^. Ca may reflect both endogenic calcites precipitating in the water column of palaeolake Chew Bahir and evaporation in the CHB basin, which is corrected for the influence of weathering in the catchment, with Ti or Zr as a clastic component^18,60,68,69^. Comparing this proxy with the total inorganic carbon and total organic carbon content of the sediment helped to correct this ratio for the presence of calcium carbonate^18^. Ti and Fe are typically found in Ti-magnetite and the volcanic bedrock in the CHB catchment (Sediment colour and colour reflectance data)^16,53,55^. Mn/Fe reflects oxic versus anoxic conditions and is closely correlated to variations in the colour reflectance values^16,18^.

### Data processing

We used MATLAB R2021a, including the Signal Processing Toolbox and the Statistics and Machine Learning Toolbox, to perform all data processing, analysis and display. All datasets (scanning and logging data) were pretreated in the following way: (1) the data files were cleaned for obvious errors; (2) data voids were replaced by the MATLAB representation for *Not-a-Number* (NaN); (3) each individual data point was flagged according to its quality (2, good data; 1, cracks and other disturbances; 0, bad data (for example, measured on plastic caps)), and all data points with flags 1 and 0 were removed from the dataset; (4) outliers were removed by using the *filloutliers* function of MATLAB, using the generalized extreme studentized deviate test for outliers; and (5) offsets due to scanning artefacts in the data series were detected using the MATLAB-based *findchangepts* function contained in the MATLAB Signal Processing Toolbox and removed by subtracting the median from the data. Before the following change-point analysis to define environmental episodes and wavelet power spectral analysis, the data were interpolated to an evenly spaced time axis with 0.1 kyr resolution.

### Definition of environmental episodes

We used a change-point analysis as one of many possible methods for structuring a climate time series such as windowed descriptive statistics^70^, wavelets^18^ or recurrence plots^25^. We again used the MATLAB-based *findchangepts* function, detecting change points^71^ by minimizing a cost function over all possible numbers and locations of change points (Extended Data Fig. 3). This function yields the number of significant changes in the mean, the standard deviation and the trend of a time series (not exceeding a maximum number of permissible changes defined by the user) that minimizes the sum of the residual error and an internal fixed penalty for each change. We run *findchangepts* for the arithmetic mean (Extended Data Fig. 3a), the standard deviation (Extended Data Fig. 3b) and the trend (Extended Data Fig. 3c) for a maximum of five significant changes. For the ~620 kyr CHB14-2 record, a limitation to a maximum of five change points was used, with a selection of infinitely more change points being possible, which would result in more and shorter episodes, respectively. Changes in the arithmetic mean, standard deviation and trend naturally occur at different places, with an overlap of a change in trend and standard deviation at ~410–411 kyr and an overlap of a change in trend and mean at ~436–437 kyr. The application of the change-point analysis on the CHB14-2 K/Zr time series yields 13 shorter and longer episodes that are each characterized by similar environmental conditions (Fig. 2).

### Wavelet power spectral analysis

We calculate a continuous wavelet transformation from the K/Zr record using the MATLAB function *cwt*. We chose *Morse* as the mother wavelet, which is very well suited to reproduce the cyclical characteristics of environmental variability in the CHB record^15,18,25^, with a symmetry parameter of 3 and a time-bandwidth product of 15. The wavelet power spectrum shows several significant periodicities that occur in certain periods but are absent in others (Extended Data Fig. 4). As an example, a clear ~100 kyr cycle appears at ~520 kyr and fades out after ~300 kyr. At about the same time, a strong ~15–25 kyr cycle appears, with a very distinct maximum centred at ~430 kyr. This maximum in the wavelet power spectrum, however, seems to be the result of two ~20 kyr cycles with extreme variations in K/Zr between 460 and 395 kyr. This interval includes an ~10 kyr episode of extremely low K/Zr values in the diagram with the *y* axis reversed between ~435 and 425 kyr, flanked by two episodes with high values at 440 and 415 kyr. A similar episode with a strong ~20–30 kyr cycle occurs between 280 and 220 kyr and an ~20 kyr cycle between 200 and 100 kyr, with a number of shorter, ~10 kyr cycles at ~175 kyr and ~125 kyr, to a lesser extent also at around ~250 kyr, and 5 kyr cycles after ~80 kyr.

## Online content

Any methods, additional references, Nature Research reporting summaries, source data, extended data, supplementary information, acknowledgements, peer review information; details of author contributions and competing interests; and statements of data and code availability are available at 10.1038/s41561-022-01032-y.

### Supplementary information

Supplementary Information Supplementary Notes 1–4, Figs. 1–3 and references.

## Figures and Tables

**Extended Data Table 1:**
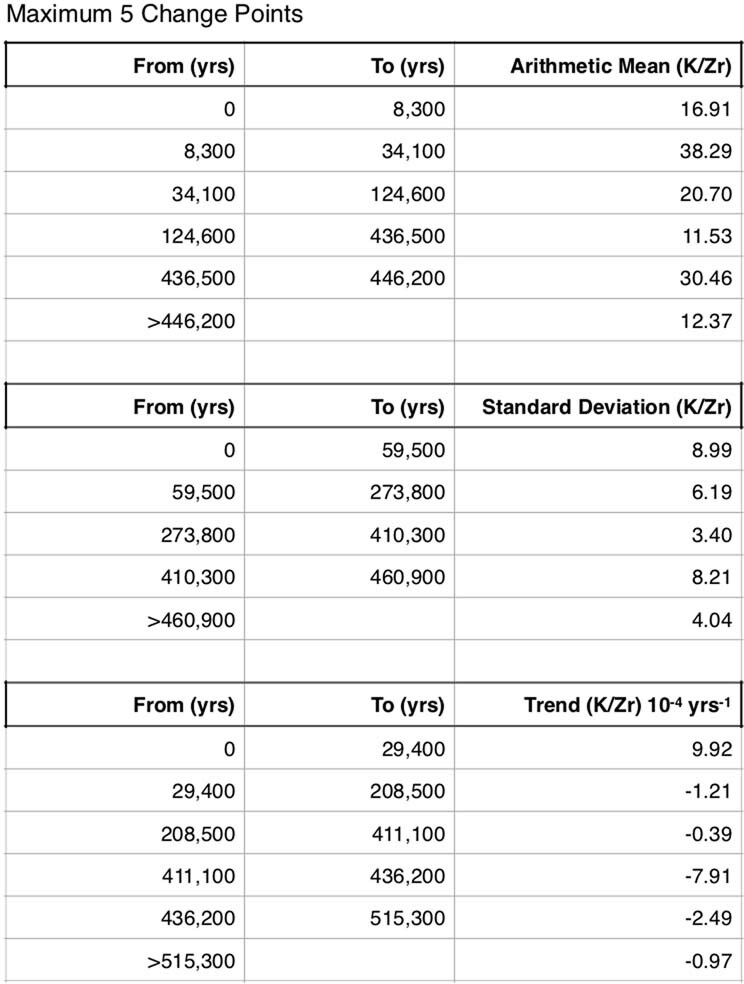
Results of the change point analysis

The change point analysis was used to define the environmental episodes, using the *findchangepts* function from the MATLAB Signal Processing Toolbox with a maximum number of five change points. The first two columns contain the intervals from the analysis as defined by the change points. The third column contains the values for the arithmetic mean, standard deviation, and trend, each in the K/Zr record of the Chew Bahir core CHB14-2.

**Extended Data Table 2:**
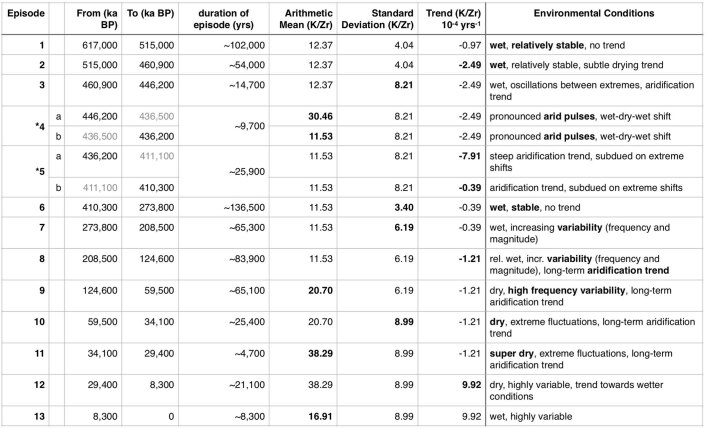
Environmental episodes in the Chew Bahir K/Zr record

Intervals as defined by the application of the results of the change point analysis (Extended Data Fig. 3 and Extended Data Table 1), duration (years), and corresponding properties in trend, arithmetic mean, and standard deviation. Last column shows inferred implications for prevailing environmental conditions during the episode.

## Data Availability

The datasets generated and analysed during this study are available online for download on the OSF repository (10.17605/OSF.IO/M8QU5). This comprises all CHB14-2 core raw data shown in Figs. 2 and 3, Extended Data Figs. 1–4, Extended Data Tables 1 and 2 and Supplementary Figures, including core metadata, core images, lithological description, splice data, stratigraphical columns, MSCL data, PC2 of colour reflectance, grain size and XRF data.

## Code availability

The data and MATLAB code to perform the change-point analysis shown in Extended Data Fig. 3 and to compute the wavelet power spectrum shown in Extended Data Fig. 4 for the K/Zr record from the CHB core are available online at http://mres.uni-potsdam.de.
