# Supplementary material for: Pleistocene climate variability in eastern Africa influenced hominin evolution
Source: Nat Geosci. 2022 Sep 26;15(10):805–11. doi: 10.1038/s41561-022-01032-y (PMC9560894; doi:10.1038/s41561-022-01032-y)
Supplement: Supplementary file 1 — Supplementary Notes 1–4, Figs. 1–3 and references. [file 41561_2022_1032_MOESM1_ESM.pdf]

nature geoscience

Article https://doi.org/10.1038/s41561-022-01032-y

Pleistocene climate variability in eastern Africa influenced hominin evolution

In the format provided by the authors and unedited

Supplementary Information for

Pleistocene climate variability in eastern Africa influenced hominin evolution

Verena Foerster[1*], Asfawossen Asrat[2,3], Christopher Bronk Ramsey[4], Erik T. Brown[5], Melissa S. Chapot[6], Alan Deino[7], Walter Duesing[8], Matthew Grove[9], Annette Hahn[10], Annett Junginger[11,12], Stefanie Kaboth-Bahr[8], Christine S. Lane[13], Stephan Opitz[14], Anders Noren[15], Helen M. Roberts[6], Mona Stockhecke[5], Ralph Tiedemann[16], Celine Vidal[13], Ralf Vogelsang[17], Andrew S. Cohen[18], Henry F. Lamb[6,19], Frank Schaebitz[1], Martin H. Trauth[8]

[1] University of Cologne, Institute of Geography Education, Cologne Germany
[2] Department of Mining and Geological Engineering, Botswana International University of Science and Technology, Palapye, Botswana
[3] Addis Ababa University, School of Earth Sciences, Addis Ababa, Ethiopia
[4] University of Oxford, Research Laboratory for Archaeology and the History of Art, Oxford, UK
[5] University of Minnesota Duluth, Large Lakes Observatory and Department of Earth & Environmental Sciences, Duluth, USA
[6] Aberystwyth University, Department of Geography and Earth Sciences, Aberystwyth, UK
[7] Berkeley Geochronology Center, Berkeley, USA
[8] University of Potsdam, Institute of Geosciences, Potsdam, Germany
[9] University of Liverpool, Department of Archaeology, Classics and Egyptology, Liverpool, UK
[10] University of Bremen, MARUM Center for Marine Environmental Sciences, Bremen, Germany
[11] Eberhard Karls Universität Tübingen, Department of Geoscience, Tübingen, Germany
[12] Senckenberg Centre for Human Evolution and Palaeoenvironment, University of Tübingen, Germany
[13] University of Cambridge, Department of Geography, Cambridge, UK
[14] University of Cologne, Institute for Geography, Cologne, Germany
[15] University of Minnesota, LacCore/CSDCO, Dept of Earth and Environmental Sciences, Minneapolis, USA
[16] University of Potsdam, Unit of Evolutionary Biology/Systematic Zoology, Potsdam, Germany
[17] University of Cologne, Institute of Prehistoric Archaeology, Cologne, Germany
[18] University of Arizona, Department of Geosciences, Tucson AZ, USA
[19] University of Dublin, Department of Botany, School of Natural Sciences, Trinity College, Dublin, Ireland

* corresponding author

This pdf file includes:

Supplementary Information text

Supplementary Note 1 for Topography, geology and climate. Chew Bahir, today a ~30 km wide and ~100 km long extensive saline mudflat at 500 m a.s.l., is a closed basin at the southern end of the Main Ethiopian Rift 4.1–6.3°N, 36.5–38.1°E) and is tectonically bounded by the >500 m high Rift escarpments to the east and west where the Chew Bahir basin is separated from the Omo-Turkana Basin by the Hammar Range (Fig. 1b)[1,2,3,4]. The western part of the 32,400 km^2 catchment is mostly formed by Late Proterozoic gneisses, whereas the eastern part is dominated by Miocene basalts[1,2,5]. The major geomorphological features at Chew Bahir are extensive alluvial fans that drain the eastern and western graben shoulders and that become periodically activated during strong rain events[1,3] Hydrologically, Chew Bahir is part of a lake-river system of closed drainage basins along the southern Ethiopian Rift system for which paleo-hydro-connectivity during pronounced pluvial phases is assumed (Lakes Abaya, Chamo and paleo-lake Chew Bahir). Chew Bahir held an up to ~45 m deep, extensive paleo-lake that is known to have overflown into Lake Turkana during pronounced wet intervals such as the African Humid Period (~15–5 ka BP) (Fig. 1b)[1,6]. The perennial Segen and Weyto Rivers (Fig. 1b) are the main sources of fluvial inwash from the north-eastern and north-western part of the catchment including the high rainfall areas of the 2500–4000 m high southwestern Ethiopian highlands (Fig. 1). The Weyto River forms a deltaic system in the northernmost part of the Chew Bahir basin, with dense vegetation, whereas towards the southern end of the basin the surface is strongly desiccated and can be subject to local aeolian deflation of fine (silty) sediments, remobilization

and sedimentation, that play however a minor role compared to the dominating fluvial-deltaic, lacustrine and authigenic processes that control the composition of the deposited sediments[1,6].

Today, rainfall in the area is associated with the seasonal migration of the tropical rain belt, resulting in bimodal rainy seasons in March-May and October-November[7]. However, Chew Bahir is adjacent to regions of unimodal and trimodal rainfall patterns, which may have shifted over the course of long-term climate change[7]. Inter-annual rainfall intensity also strongly depends on Atlantic and Indian Ocean sea-surface temperature (SST) variations associated with the Indian Ocean Dipole and the El Niño-Southern Oscillation[8].

Supplementary Note 2 for radiometric age determination and age-depth modeling. We have developed two independent age models for the composite core HSPDP-CHB14-2 (Supplementary Fig. S1). For the K/Zr time series discussed here, we applied age model RR2021[9] that is based on multiple independent chronometric techniques using radiocarbon dating of ostracods, optically-stimulated luminescence (OSL) dating of quartz, single-crystal total-fusion (SCTF) $^{40}Ar/^{39}Ar$ dating of K-feldspars from tuffaceous zones, and geochemical correlation of the Konso Silver Tuff (Silver Vitric Tuff, SVT) to a visible tephra unit in the core[9,10]. Applying RR2021, the 5 mm spacing of the K/Zr resolution corresponds to a temporal resolution of ~10 years. The 30 ages generated by these chronometric techniques are stratigraphically consistent; Bayesian age-depth modeling incorporating the ^{14}C, OSL and $^{40}Ar/^{39}Ar$ ages, and tephrochronological data, has been used to build an age-depth model for the composite core[9]. The Bayesian age-depth model takes into account the potential for hiatuses and variations in sedimentation rates such as may naturally occur during wet-dry cycles, producing modelled ages with uncertainties throughout the sedimentary sequence. Whilst the mean of the Bayesian age model output effectively linearly interpolates between the sparser data-points in the earliest parts of the core, the uncertainties generated by the model do still cover possible hiatuses and variation in sedimentation rate, averaging across all such possibilities[9]. The 1-sigma absolute uncertainties produced, increase with increasing depth, ranging from <10 ka in the uppermost 50 m (corresponding to ~100 ka BP) and 10–35 ka below 50 m composite depth (~100–620 ka BP).

An alternative age model MUBAWA2021 uses the multiband wavelet age modeling (MUBAWA) algorithm that tracks the earth's precession cycle in the wavelet power spectrum of the MSCL-based sediment-color reflectance values[11]. We first use a principal component analysis (PCA) to separate variations of blue-green colors (during wet episodes) to reddish-brown colors (during dry episodes) (stored in the 2nd principal component PC2) from the total reflectance (or brightness) of the sediment (PC1) in sediment-color values. The climatically-controlled color variations recorded in the PC2 show distinct cycles with wavelengths of 10–15 m and of ~40 m in the core, probably a result of the influence of the earth's precession and eccentricity cycles[11]. We then use an adaptive bandpass filter to extract the approximate spatial wavelength range from the sediment color data, which corresponds to the wavelength of our tuning target, the earth's precession cycle according to Laskar et al. (2004) [11,12].

Comparing RR2021[9] and MUBAWA2021[11] reveals that both independent age models agree well with each other, particularly in the deepest parts of the core where they also agree within uncertainties with evidence from direct dating provided by four $^{40}Ar/^{39}Ar$ ages (Suppl. Fig. S1). A closer look at the plot of the differences between the modeled ages, reveals that the maximum differences between the two age models accounts for one and a half precession cycles (Suppl. Figs. S1 and S2). These differences between the models are greatest for the upper portion of the core where the direct dating evidence that underpins the Bayesian model RR2021 is the most abundant (e.g. 26 of the 30 radiometric ages from multiple direct dating methods are located within the upper 75m of the core). Below 75 m, only four $^{40}Ar/^{39}Ar$ ages are available and the RR2021 model is less well constrained, while the MUBAWA2021 model is calibrated to the precession cycle and therefore provides more detail for this interval. Nevertheless, from ~150 m composite depth through to the base of the core, which spans more than 300 ka duration, the RR2021 and MUBAWA2021 models are in particularly close agreement (Suppl. Fig. S1).

To avoid the potential circularity of using an orbitally-tuned age model to investigate a potential proxy for climate, particularly when a robust age model based on multiple direct dating techniques exists, the Bayesian model RR2021[9]is used to provide the chronology for the K/Zr record presented in this study. Exploring the detailed differences between the XRF-based (K/Zr) record adjusted to the RR2021[9] and MUBAWA2021[11] age models (Suppl. Fig. S2 illustrates the previously noted offsets, but shows that the general long-term trends are in good agreement, and the differences between them lie within their uncertainties. Because of the uncertainties in our chronology, and especially because of greater uncertainties in the comparative archives, our interpretation and comparisons of transitions in this record with those in other climate archives is undertaken with great caution.

Supplementary Note 3 for Forcing. Forcing and SST variations: A second modifier of hydroclimatic change in Chew Bahir is sea-surface temperature (SST) variations of the surrounding oceans with particularly the Indian Ocean as the main humidity source for eastern Africa[7,13,14,15]. In addition to the insolation effect on air mass transport controlled by the land-sea temperature gradients, higher SSTs can enhance temperature-dependent evaporation over the ocean and therewith strongly influence the amount of water in the troposphere. A weakening of low-level winds along the eastern coast of Africa and across the Arabian Sea during long-term insolation driven phases of warmer ocean temperatures, had dampening effects on upwelling, once again enhancing convergence over the western equatorial Indian Ocean[16]. Thus the CHB long-term wet-dry oscillations show generally a strong similarity to SST fluctuations recorded in ODP 722[17], that reflect the intensity of coastal upwelling in the Arabian Sea, and thus the strength of the summer monsoon[18,19], suggesting that amplified wet phases in southern Ethiopia might have been caused by enhanced moisture advection and a stronger summer monsoon in phase with orbital forcing (Suppl. Fig. S3).

Forcing and glacial boundary conditions: In addition to insolation forcing and SST changes of the neighboring Indian Ocean, atmospheric CO_2 variability has also been proposed as a driver of tropical climate change throughout the geological past[20]. Our comparison of the Antarctic EPICA CO_2[21] record with CHB moisture fluctuations suggests, however, that greenhouse gases only played a minor role for the moisture budget of CHB with possible exceptions during the eccentricity minimum phases between ~430–360 ka and ~120–0 ka, respectively. Here, the lowered insolation levels appear to coincide with distinct moisture phases at the transition into interglacial conditions (Suppl. Fig. S3). The forcing of large ice sheets however could have played a role for precipitation in eastern Africa during muted orbital forcing, when under glacial conditions and lowered greenhouse gas concentrations convection was generally reduced in the near-equatorial zones and westerly controls weakened monsoons and stormtracks[22,23]. This is in agreement with model simulations considering orbital, greenhouse gas, and ice sheet forcing of the moisture variability over northern Africa and the Arabian peninsula suggesting that during interglacial conditions the variations between orbitally forced wet/dry extremes has seen some of the wettest phases due to the increase of NH monsoons in addition to increased winter rains from the Mediterranean basin. On the other hand a decreased NH seasonality coincided with weakened monsoons and precipitation decrease from the Mediterranean storm tracks[22]. This is particularly well expressed at ~430 ka and the transition into MIS 11, and could potentially relate to the simultaneous Mid-Bruhnes Event (MBE). Although the origin of the MBE is not completely understood, it was associated with persistently enhanced interglacial CO_2 levels during the late mid-Pleistocene[24]. In turn, increased CO_2 levels might have resulted in enhanced annual and seasonal rainfall rates, and increased extreme wet period frequency as predicted for eastern African climate under current climate change.

Supplementary Note 4 on the Categorization of dimensions in cultural innovation. Important developments in cultural and technological innovation have been proposed to be bundled in eight grades that describe the expansion of cultural capacity (EECC model) by Haidle et al. (2015)[25] (Fig. 3). Each onset of these phases is represented by "minimum ages", i.e. the oldest certain evidence. Stage 6, from <200 ka on, encompasses the development of critical cognitive abilities that enable humans to plan and execute technological augmentations such as composite tools as found in hafted tools and compound adhesives[25–29]. It has been argued that this key set of innovation also represents an important expansion of cultural capacity[27]. The onset of Stage 7, from 64 ka on, is marked by a notable advance in complementary cultural capacity, that entails the development and use of a set of cultural modules as an acting entity with two or

more interdependent and exchangeable parts. An example would be the emergence of bow-and-arrow, needle-and-thread, screw- and screwdriver, key-and-lock[26,30]. Earliest evidence for the emergence of a notional cultural capacity, Stage 8, appears around ~40 ka[31]. The development of a notional cultural capacity contains that notional concepts are mentally constructed and socially shared entities and relationships that can be represented in a) the signification of objects/signs (e.g. cross, crescent, and Star of David as symbols of religions), b) systems of ideas (e.g. myths, religious beliefs, philosophical questions, constitutions of states), c) normative definitions (e.g. metric and value systems), or d) virtual beings (e.g. angels) and characters (e.g. protecting capacities of an amulet)[25].

The transport of raw material over longer distances can be used as a key indicator for the formation of larger social networks. As early as ~320 ka ago, obsidian has been shown to be transported and exchanged in Olorgesailie (Kenya) over a distance of ~50 km, whereas evidence from Baringo (Kenya) ~200 ka ago indicates already a much larger network radius of 166 km[31–33].

Supplementary References

1. Foerster, V. et al. Climatic change recorded in the sediments of the Chew Bahir basin, southern Ethiopia, during the last 45,000 years. *Quaternary International* **274,** 25–37 (2012).
2. Davidson, A. The Omo River Project: Reconnaissance Geology and Geochemistry of Parts of Ilubabor, Kefa, Gemu Gofa and Sidamo. *Ethiopian Institute of Geological Surveys Bulletin* **2**, 1–89 (1983).
3. Gebregiorgis, D. et al. Modern Sedimentation and Authigenic Mineral Formation in the Chew Bahir Basin, Southern Ethiopia: Implications for Interpretation of Late Quaternary Paleoclimate Records. *Front. Earth Sci.* **9:607695**, 1–18, doi: 10.3389/feart.2021.607695 (2021).
4. Schaebitz, F. et al., Hydroclimate changes in eastern Africa over the past 200,000 years may have influenced early human dispersal. *Nature Communications Earth and Environment* **2**, 1–10 (2021).
5. Foerster, V. et al. Towards an understanding of climate proxy formation in the Chew bahir basin, southern Ethiopian Rift. *Palaeogeogr. Palaeoclimatol. Palaeoecol.* **501**, 111–123 (2018).
6. Fischer, M.L. et al. Determining the Pace and Magnitude of Lake Level Changes in Southern Ethiopia Over the Last 20,000 Years Using Lake Balance Modeling and SEBAL. *Frontiers in Earth Science* **8**, http://doi.org/10.3389/feart.2020.00197 (2020).
7. Nicholson, S.E. Climate and climatic variability of rainfall over eastern Africa. *Reviews of Geophysics* **55**, 590–635 (2017).
8. Viste, E. & Sorteberg, A. The effect of moisture transport variability on Ethiopian summer precipitation *Int. J. Climatol.* **33,** 3106–3123 (2013).
9. Roberts, H.M. et al. Using multiple chronometers to establish a long, directly-dated lacustrine record: constraining >600,000 years of environmental change at Chew Bahir, Ethiopia. *Quaternary Science Reviews* **266**, 1–17 (2021).
10. Clark, J.D. et al. Stratigraphic, chronological and behavioural contexts of Pleistocene *Homo sapiens* from Middle Awash, Ethiopia. *Nature* **423**, 747–752 (2003).
11. Duesing, W. et al. Multiband Wavelet Age Modeling for a 293 m (600 kyr) Sediment Core From Chew Bahir Basin, Southern Ethiopian Rift. *Front. Earth Sci.* **9,** 594047. doi: 10.3389/feart.2021.594047 (2021).
12. Laskar, J. et al. A long term numerical solution for the insolation quantities of Earth. *Astronomy Astrophys.* **428**, 261–285 (2004).
13. Nicholson, S.E. The ITCZ and the Seasonal Cycle over Equatorial Africa. *Bulletin of the American Meteorological Society* **99**, 337–348 (2018).
14. Jalihal, C. et al. The response of tropical precipitation to Earth's precession: the role of energy fluxes and vertical stability. *Clim. Past* **15**, 449–462 (2019).
15. Kaboth-Bahr, S. et al. Paleo-ENSO influence on African environments and early modern humans. *PNAS* **118**, e2018277118 (2021).
16. deMenocal P.B. Plio-Pleistocene African climate. *Science* **270**, 53–59 (1995).

17. Herbert, T., Cleaveland Peterson, L., Lawrence, K.T. & Liu, Z. Tropical Ocean Temperatures Over the Past 3.5 Million Years. *Science* **328**, 1530–1534 (2010).

18. Clemens, S.C. & Prell, W.L. One million year record of summer monsoon winds and continental aridity from the Owen Ridge (Site 722), northwest Arabian Sea. In: Prell, W.L. et al. (eds.) *Proceedings of the Ocean Drilling Program*, Scientific Results, College Station, TX (Ocean Drilling Program) **117**, 365–388 (1991).

19. Lüthi, D. et al. High-resolution carbon dioxide concentration record 650,000–800,000 years before present. *Nature* **453**, 379–382 (2008).

20. Clemens, S., Prell, W., Murray, D., Shimmield, G. & Weedon, G. Forcing mechanisms of the Indian Ocean monsoon. *Nature* **353**, 720–725 (1991).

21. Bereiter, B. et al. Revision of the EPICA Dome C CO2 record from 800 to 600 kyr before present. *Geophysical Research Letters* **42**, 542–549 (2015).

22. Trauth, M.H., et al. High- and low-latitude forcing of Plio-Pleistocene East African climate and human evolution. *Journal of Human Evolution* **53**, 475–486 (2007).

23. Kutzbach, J. E., & Street-Perrott, F. A. Milankovitch forcing of fluctuations in the level of tropical lakes from 18 to 0 kyr BP. Nature **317**, 130–134 (1985).

24. Owen, R.B. et al. Progressive aridification in East Africa over the last half million years and implications for human evolution. *Proceedings of the National Academy of Sciences* **115**, 11174–11179 (2018).

25. Haidle, M.N. et al. The Nature of Culture: an eight-grade model for the evolution and expansion of cultural capacities in hominins and other animals. J Anthropol Sci 93, 43–70 (2015).

26. Lombard, M. & Haidle, M.N. Thinking a bow-and-arrow: cognitive implications of Middle Stone Age bow and stone-tipped arrow technology. Camb. Archaeol. J. 22, 237–264 (2012).

27. Wynn, T., Coolidge, F. & Bright, M. Hohlenstein-Stadel and the evolution of human conceptual thought. *Camb. Archaeol. J.* **19,** 73–83 (2009).

28. Barham, L. From hand to handle: the first industrial revolution. Oxford, UK: Oxford University Press. (2013).

29. Wadley, L. Compound-adhesive manufacture as a behavioral proxy for complex cognition in the Middle Stone Age. *Curr. Anthropol.* **51**, 111–119 (2010).

30. Williams V.M.E., Burke A. & Lombard M. Throwing spears and shooting arrows: preliminary results of a pilot neuroarchaeological study. *S. Afr. Archaeol. Bull.* **69**, 199–207 (2014).

31. Barton, R.N.E. et al. OSL dating of the Aterian levels at Dar es-Soltan I (Rabat, Morocco) and implications for the dispersal of modern Homo sapiens. *Quaternary Science Reviews* **28,** 1914-1931 (2009).

32. Blegen, N. The earliest long-distance obsidian transport : evidence from the ~ 200 ka middle stone age sibilo school road site, Baringo, Kenya. *J. Hum. Evol.* **103**, 1–19 (2017).

33. Deino, A.L. et al. 2018. Chronology of the Acheulean to Middle Stone Age transition in eastern Africa. *Science* **360**, 95–98 (2018).

34. Potts, R. et al. Environmental dynamics during the onset of the Middle Stone Age in eastern Africa. *Science* **360,** 86–90 (2018).

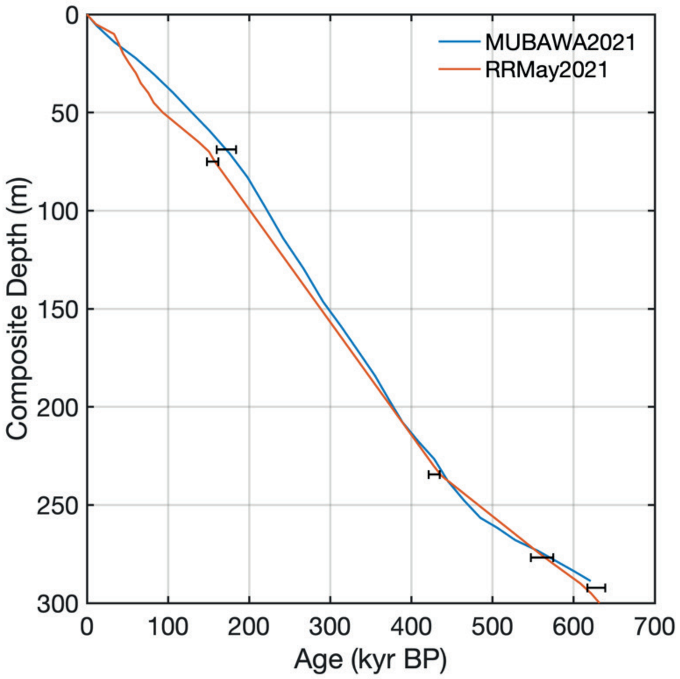

Supplementary Figure S1 I Comparison of available age-depth models for the composite core HSPDP-CHB14-2. The simplified output from the Bayesian age-depth model RR2021(Roberts et al., 2021) (red line) is based on direct dating using multiple independent chronometric techniques, and the MUBAWA2021 (Duesing et al., 2021) model (blue line) uses the multiband wavelet age modeling technique to derive a chronology by tuning the Chew Bahir record to astronomical precession.

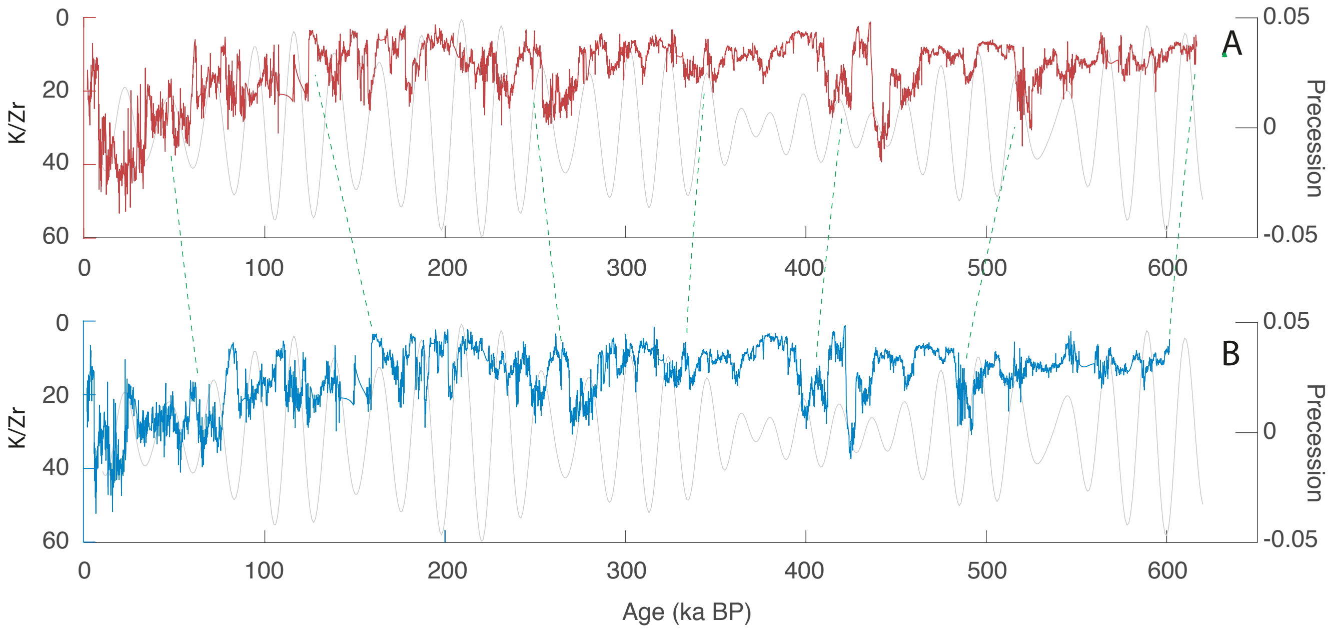

Supplementary Figure S2 | Comparing the XRF-based CHB14-2 (K/Zr) record adjusted to the (A) RR2021 (Roberts et al., 2021) (red line) and (B) MUBAWA2021 (Duesing et al., 2021) (blue line) age models.

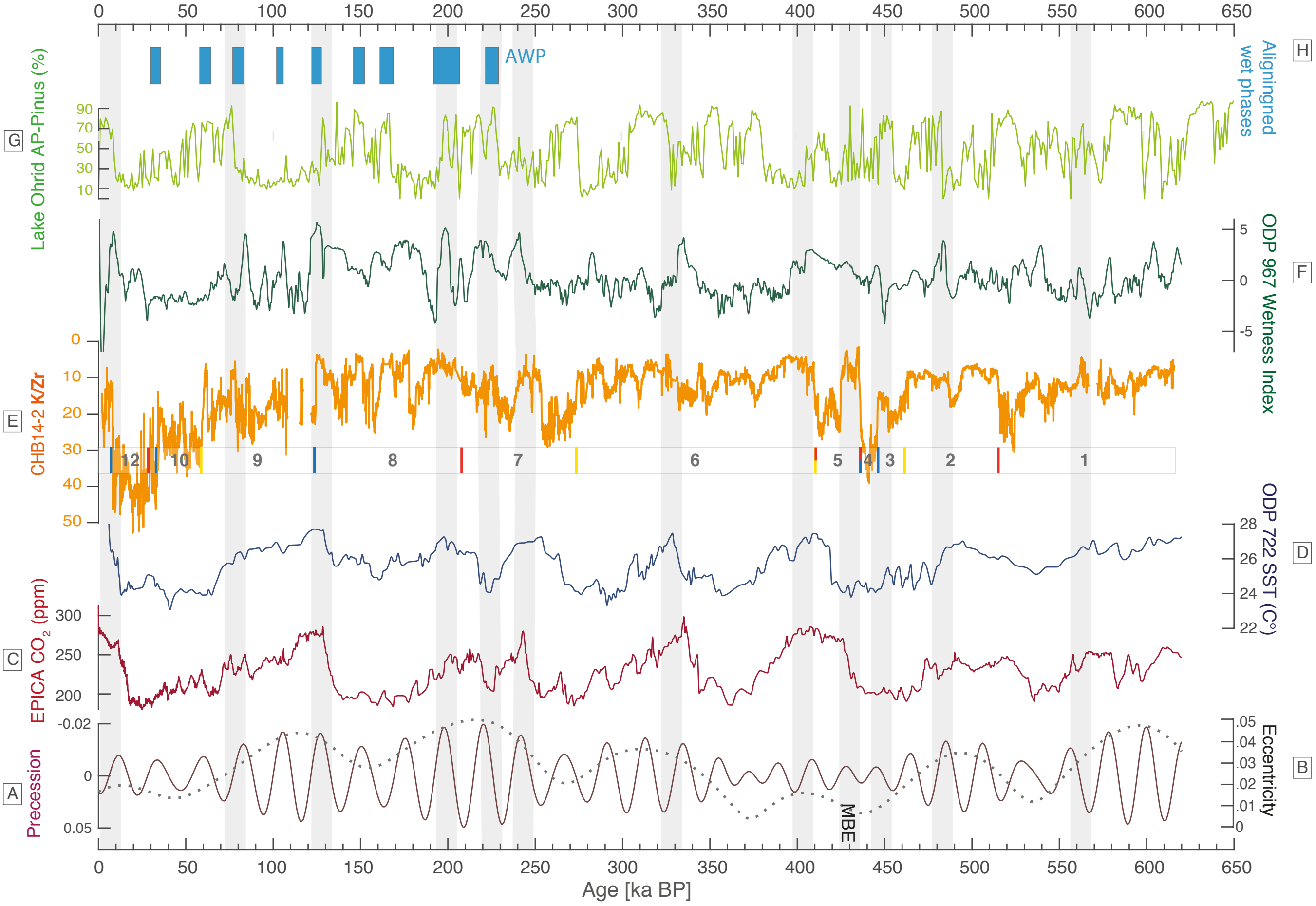

Supplementary Figure S3 | Comparison of the Chew Bahir record with proposed controls of climate change and other paleoclimate records. Records from bottom to top: orbital forcing with (A) precession (Laskar et al., 2004) and (B) eccentricity (Laskar et al., 2004), (C) atmospheric CO_2 (ppm) from EPICA Dome C, Antarctica (Bereiter et al., 2015), (D) SST variations (C°) from ODP site 722 in the Arabian Sea (Herbert et al., 2010), (E) Chew Bahir CHB14-2 K/Zr aridity indicator (this study, note inverted scale), southern Ethiopia (F) wet-dry index from ODP site 967 in the eastern Mediterranean Sea (Grant et al., 2017), (G) AP-Pinus pollen record from Lake Ohrid (Wagner et al., 2019), (H) aligned wet phases (AWP), co-occurring in CHB (eastern Africa) and Lake Ohrid (NE-Mediterranean) and NE-Africa. MBE = Mid-Brunhes Event. The CHB14-2 proxy records are based on the Bayesian age model RR2021 (Roberts et al., 2021).
